# An Innovative Technique of Revision Surgery for Distal Junctional Failure

**DOI:** 10.7759/cureus.87802

**Published:** 2025-07-12

**Authors:** Masato Tanaka, Savvas Moschos, Chen B Jein, Aman Verma, Mohammed A Rezk Sharaf E H

**Affiliations:** 1 Department of Orthopedic Surgery, Okayama Rosai Hospital, Okayama, JPN; 2 Department of Orthopedic Surgery, Okayama University Hospital, Okayama, JPN

**Keywords:** c-arm free, distal junctional failure, navigation, reoperation, transdiscal screw

## Abstract

Distal junctional kyphosis (DJK) or the adding-on phenomenon is one of the most challenging complications following long fusion for adolescent idiopathic scoliosis (AIS) or adult spinal deformity (ASD). Among these complications, distal junctional failure (DJF) is defined as a condition requiring revision surgery due to severe symptoms such as intense low back pain, myelopathy, and difficulty in standing and walking. Three patients developed severe lower back pain due to DJF, and two patients underwent revision surgery with a new technique. Surgical outcomes, surgical time, intraoperative blood loss, and operative complications were evaluated. Two patients underwent revision surgery with a novel method for revising distal screw loosening without excessive distal extension. There was no complication, and two patients had solid bony fusion at a two-year follow-up. The revision surgery for type 1 DJK can be challenging because the conventional pedicle screw technique is not feasible for the lower instrumented vertebra, and a more extended distal fusion is required. The adoption of O-arm-guided transdiscal screw fixation has significant clinical implications. This technique also increases accuracy in screw placement, mitigating the risks associated with traditional revision methods and preserving motion segments.

## Introduction

Distal junctional kyphosis (DJK) is a condition where either (i) The forward curvature (kyphosis) at the lowest part of the spinal fusion (between the last fused vertebra and the one below it) is greater than 10°, or (ii) There is an increase of more than 5° in the forward bend (kyphotic change) between the last fused vertebra (LIV) and the vertebra below it (LIV+1) after surgery or during follow-up [1]. DJK is not rare in association with adult spinal deformity (ASD) [2] and Scheuermann disease [3]. However, distal junctional failure (DJF) is identified through clinical and radiographic signs of instability at the lower end of the spinal construct. The adding-on phenomenon is a complication that can occur after fusion surgery, especially scoliosis surgery. It's characterized by a loss of correction and an increase in the number of vertebrae in the distal curve [4]. When revision surgery is necessary for these conditions due to severe symptoms such as intense low back pain, myelopathy, and difficulty standing and walking, those conditions are regarded as DJF [5]. The causes of DJF are fracture of the lower instrumented vertebra, failure of the instrumentation at the most distal level (screw backout, loosening, rod or screw breakage), spinal stenosis/instability, and pseudoarthrosis [6].

For mechanical failures such as screw backout or loosening of the vertebra, conventional pedicle screws are less effective due to the previous void created by a loosened large screw inside the vertebra, which compromises the purchase for instrumentation. Revision surgery can be challenging and often requires extending the fusion area distally. However, preserving the motion segment in the lumbar spine is crucial for patients to maintain a normal lifestyle and prevent further degenerative joint disease. To achieve strong purchase for a previously instrumented void vertebra, there are several options, including the use of large screws, augmented screws [7], cortical bone trajectory screws [8], and extended fixation. In many cases, these techniques are not suitable. The authors hereby present a novel technique utilizing transdiscal screws to achieve a strong anchor for the haled vertebra, thereby preventing long fusion as a DJF revision surgery of two cases.

## Technical report

Surgical technique

**Figure 1:**
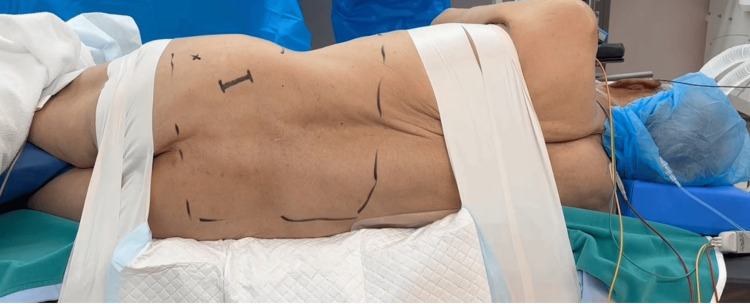
Patient positioning

**Figure 2:**
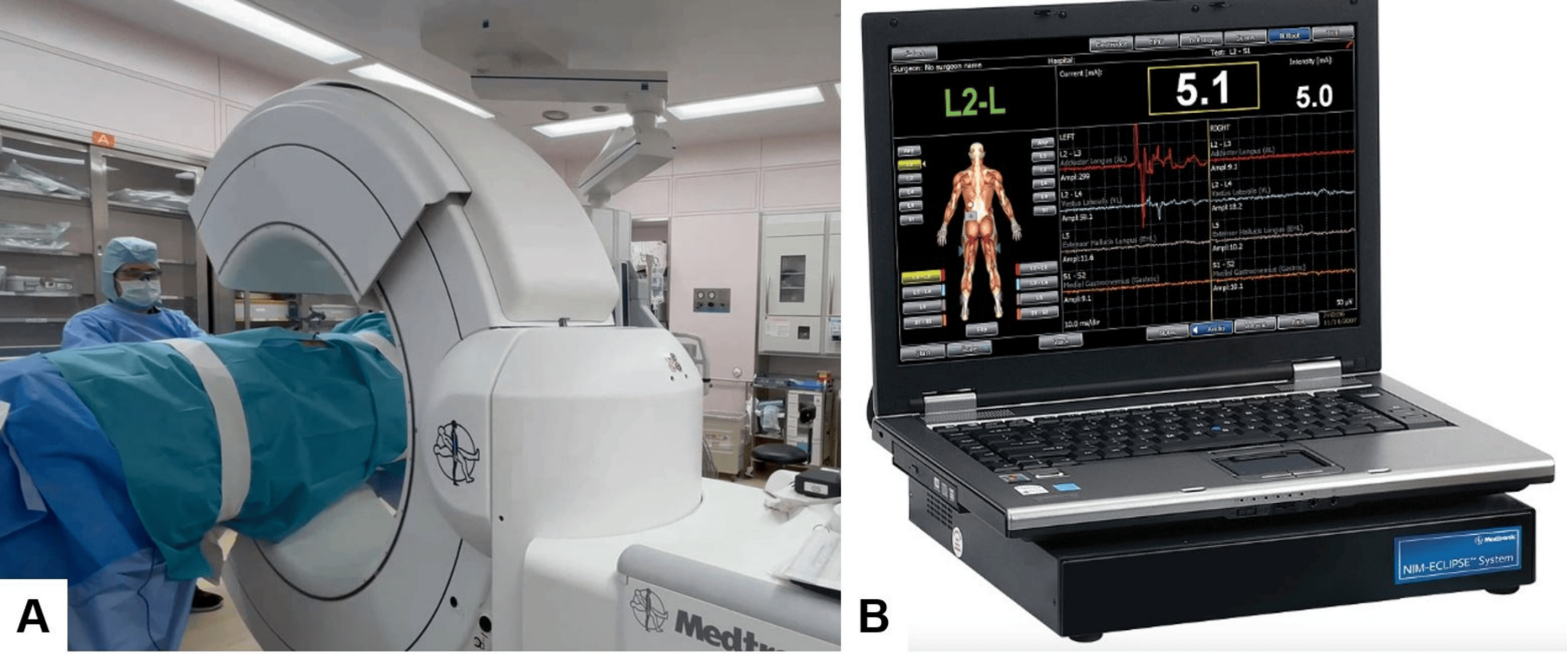
Operating room setup A: O-arm and B: Neuromonitoring.

**Figure 3:**
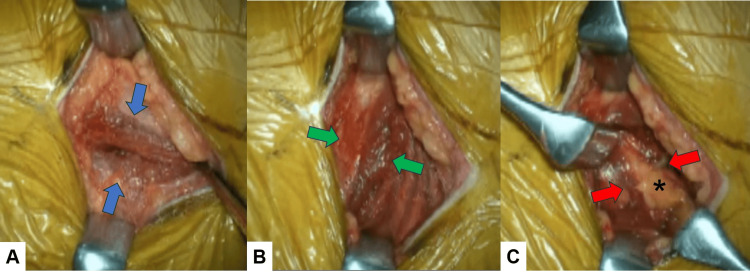
Muscle dissection A: Blue arrows indicate the external oblique abdominal muscle, B: Green arrows show the internal oblique abdominal muscle, and C: Red arrows indicate the transverse abdominal muscle. * indicates epidural fat tissue.

**Figure 4:**
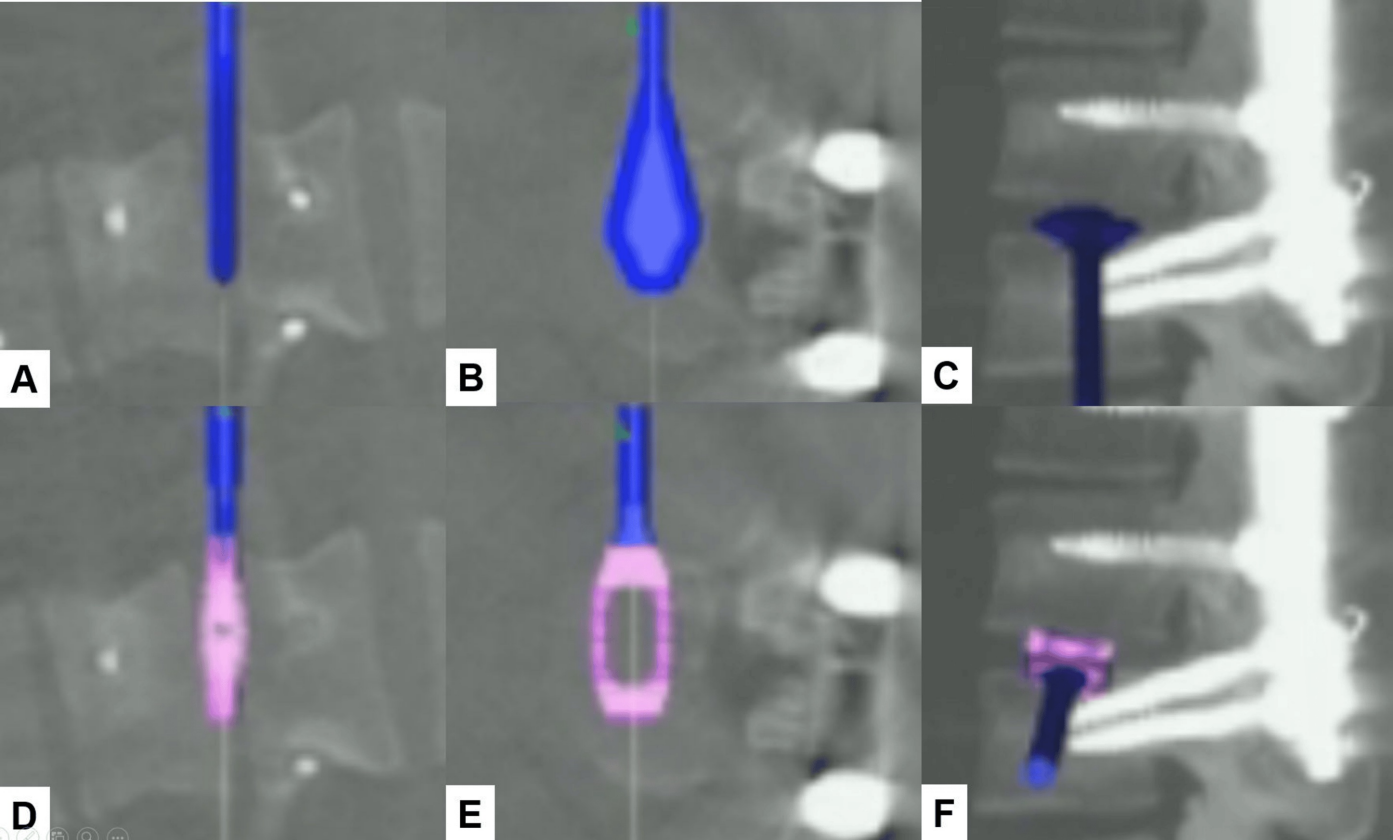
Trial and case insertion A: Coronal image of a trial, B: Axial image of a trial, C: Sagittal image of a trial, D: Coronal image of a cage, E: Axial image of a cage, and E: Sagittal image of a cage. The disc space was carefully expanded using a navigated spreader, and the navigated cage was inserted under navigation guidance.

The procedure is performed in a right lateral decubitus position and with neuromonitoring (Figure 1). This method with O-arm navigation avoided the use of a C-arm, thereby minimizing radiation exposure. Continuous neuromonitoring was initiated at the start of the procedure to ensure the patient’s neurological safety throughout the procedure. Following sterilization and draping, a reference frame was carefully inserted into the left sacroiliac joint. A CT scan was then obtained using the O-arm to provide detailed imaging guidance (Figure 2). A precise 4 cm lateral skin incision was made, guided by a navigational probe, according to the fusion level. This incision allowed for the sequential dissection of the external, internal, and transverse abdominal muscles, providing access to the surgical site (Figure 3). After registering each surgical instrument, the intended lumbar disc spaces were accurately exposed with the aid of a navigation probe. A self-retaining retractor with illumination was then placed to maintain clear visibility and access to the operative field. Discectomy at the disc level was meticulously performed using a knife and navigated shavers, followed by complete disc removal with a knife and navigated shavers followed by complete disc removal with a navigated Cobb elevator. Subsequently, the disc space was carefully expanded using a navigated spreader and trial (Figures 4A-4C). Finally, the navigated cage was inserted under navigation guidance (Figures 4D-4F).

**Figure 5:**
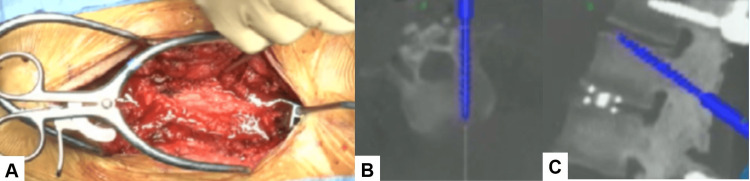
Pedicle screw insertion A: Intraoperative image, B: Axial image, and C: Sagittal image. Transdiscal screws should be aimed toward the upper endplate to penetrate the superior endplate.

**Figure 6:**
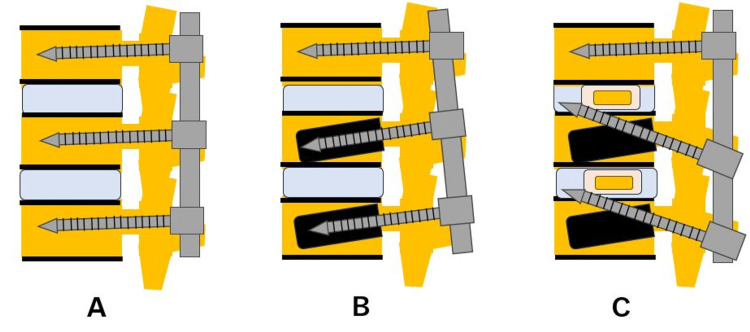
Schema of the new revision technique for DJK A: No screw loosening, B: Screw loosening, and C: Revision surgery using transdiscal screws. These transdiscal screws were across the previous screw hole and enhanced the screw pullout strength. DJK: Distal junctional kyphosis

In a prone position, transdiscal screws should be aimed toward the upper endplate to penetrate the superior endplate of the working level (approximately 25 degrees). These transdiscal screws are relatively strong because these screws penetrate sclerotic previous screw hole walls twice and the upper endplate of the vertebra (Figures 5, 6). 

Case 1

**Figure 7:**
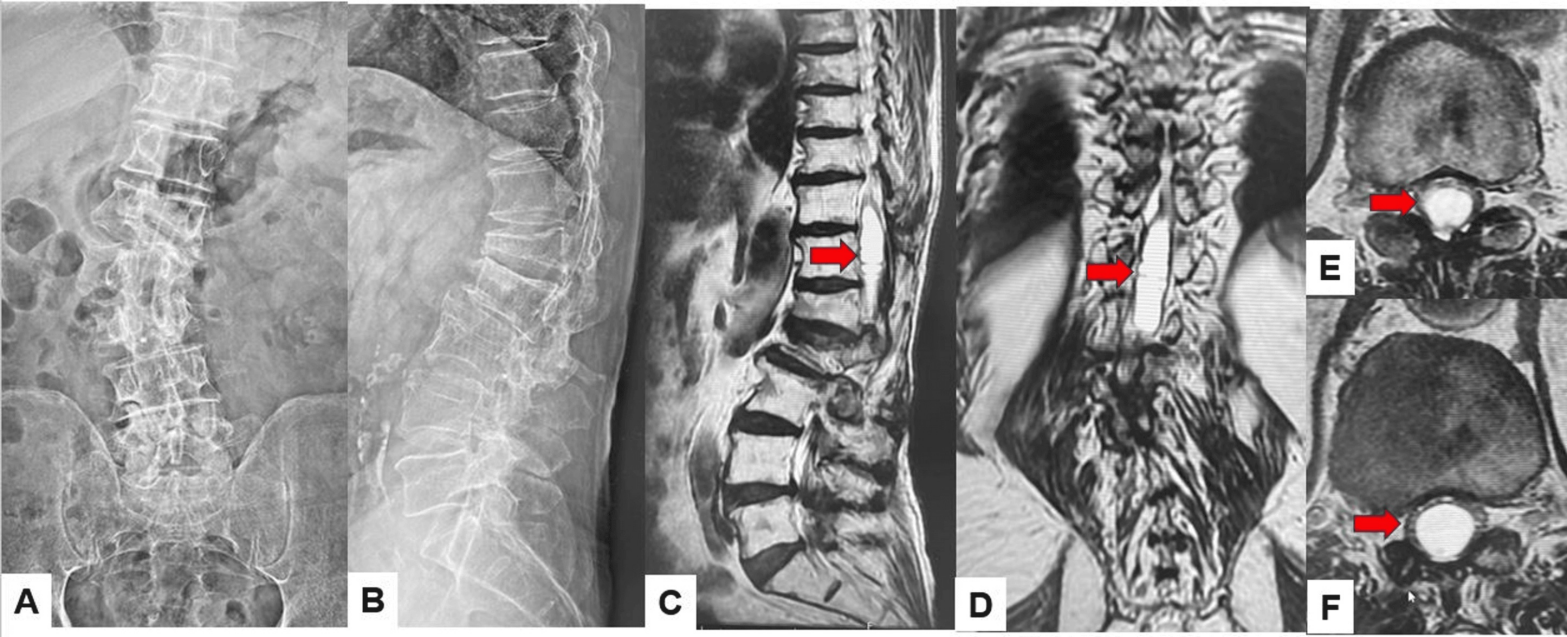
Preoperative images A: Anteroposterior lumbar radiogram, B: Lateral lumbar radiogram, C: T2-weighted mid sagittal lumbar MRI, D: T2-weighted coronal lumbar MRI, E: T2-weighted axial MRI at T12, and F: T2-weighted axial MRI at T12/L1. Red arrows indicate a large syringomyelia.

**Figure 8:**
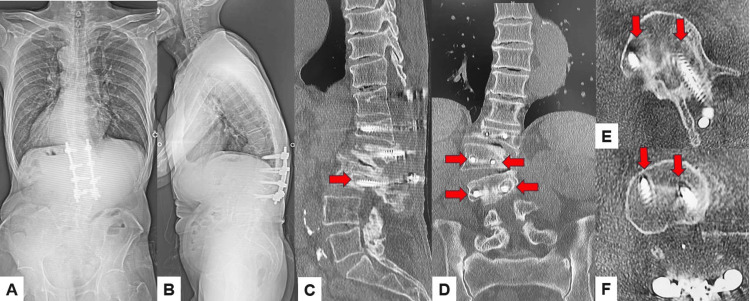
Postoperative images A: Posteroanterior whole spinel radiogram, B: Lateral whole spine radiogram, C: Midsagittal lumbar reconstruction CT, D: Coronal lumbar reconstruction CT, E: Axial CT at L2, and F: Axial CT at L3. Red arrows indicate screw loosening.

**Figure 9:**
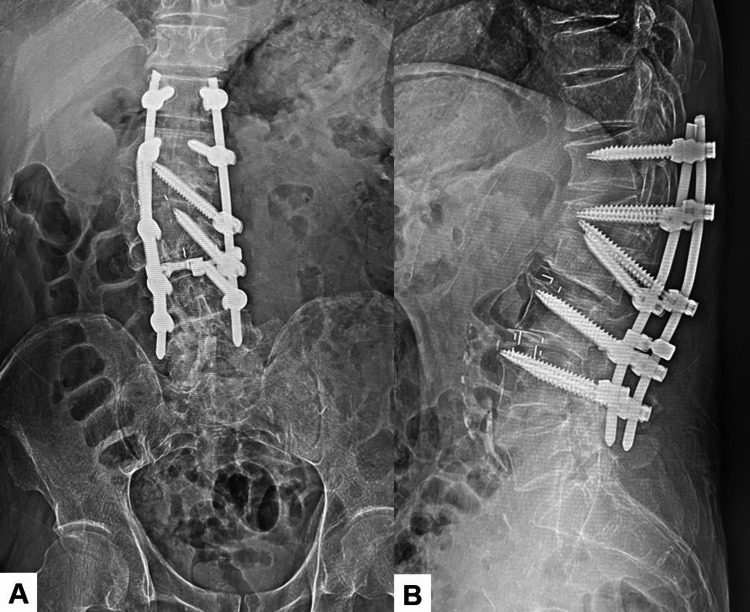
Post-revision radiogram A: Anteroposterior lumbar radiogram and B: Lateral lumbar radiogram.

**Figure 10:**
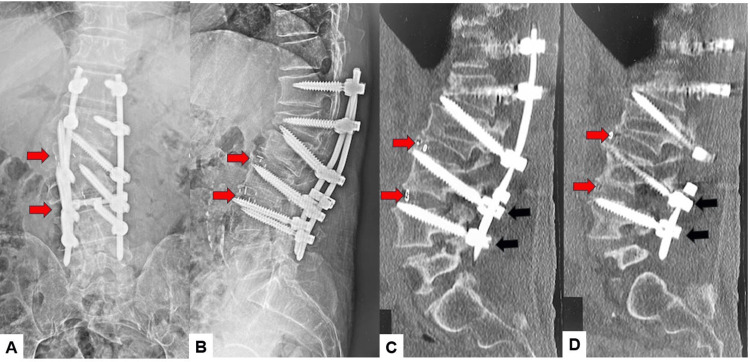
Final follow-up images A: Anteroposterior lumbar radiogram, B: Lateral lumbar radiogram, C: Left parasagittal lumbar reconstruction CT, and D: Right parasagittal lumbar reconstruction CT. Red arrows indicate OLIF cages. Block arrows showed transdiscal screws. OLIF: Oblique lateral interbody fusion

Case 1 presents a type 1 DJF, 72-year-old man with adult spinal deformity and syringomyelia. A 72-year-old man was referred to our hospital due to bilateral leg weakness. He had had a thoracolumbar fracture due to a traffic accident. He cannot walk without a cane. Manual muscle testing of the bilateral legs was 4/5. He had bilateral numbness of both legs and urinary disturbance. Preoperative radiogram showed L1 burst fracture and thoracolumbar scoliosis. Preoperative MRI indicated large syringomyelia at the thoracolumbar area (Figure 7). The patient underwent subarachnoid-subarachnoid shunting and thoracolumbar posterolateral fixation with the pedicle screw system. After three months of this surgery, his low back pain was increased due to distal screw loosening (Figure 8). The revision surgery was undergone using transdiscal screws and oblique lumbar interbody fusion (Figure 9). Those transdiscal screws were across the previous screw hole and enhanced the screw pullout strength. The final radiogram showed solid bony fusion and no screw loosening (Figure 10).

Case 2

**Figure 11:**
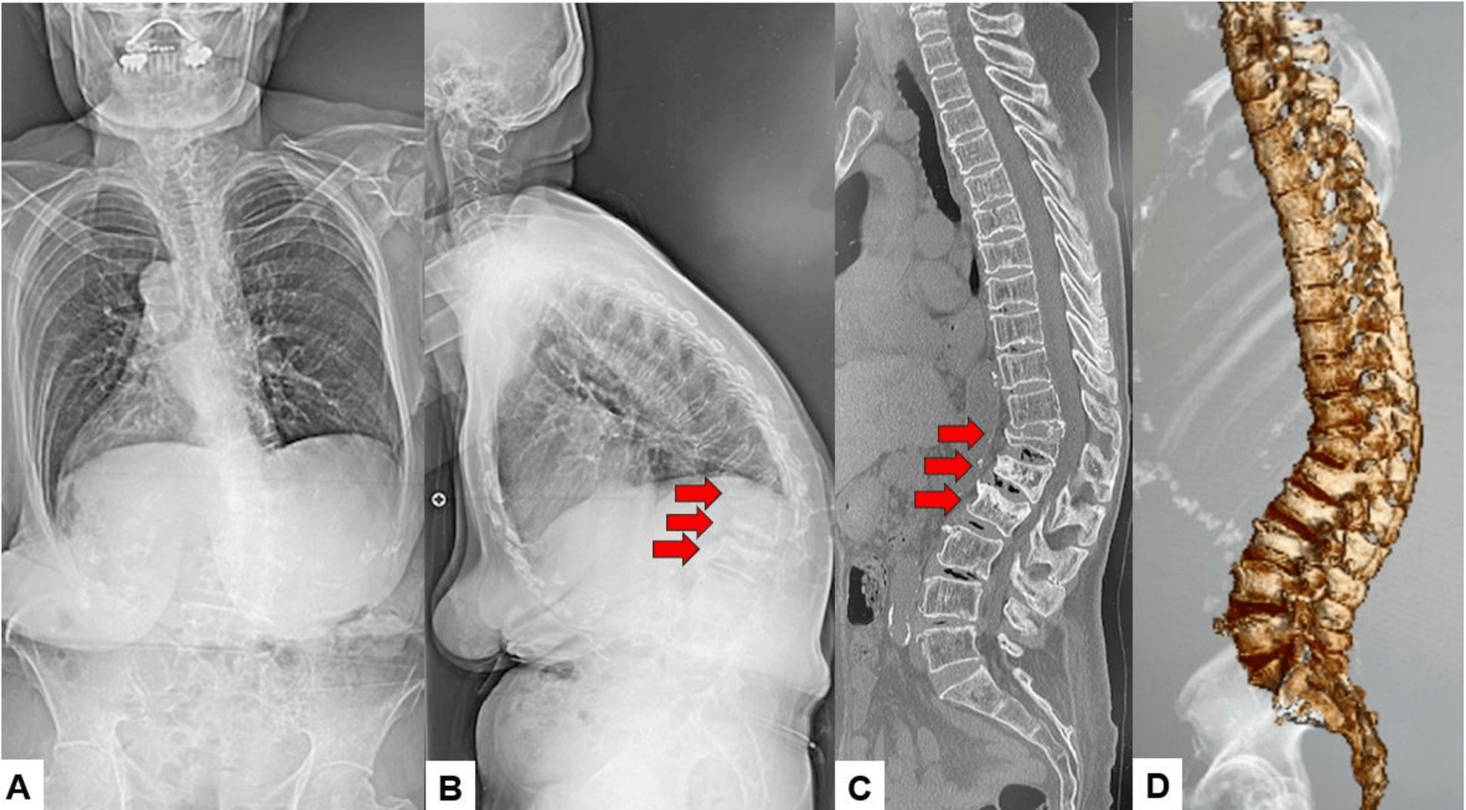
Preoperative radiograms A: Posteroanterior whole spine radiogram, B: Lateral whole spine radiogram, C: Mid sagittal reconstruction CT, and D: 3D CT. Red arrows indicate osteoporotic vertebral fractures.

**Figure 12:**
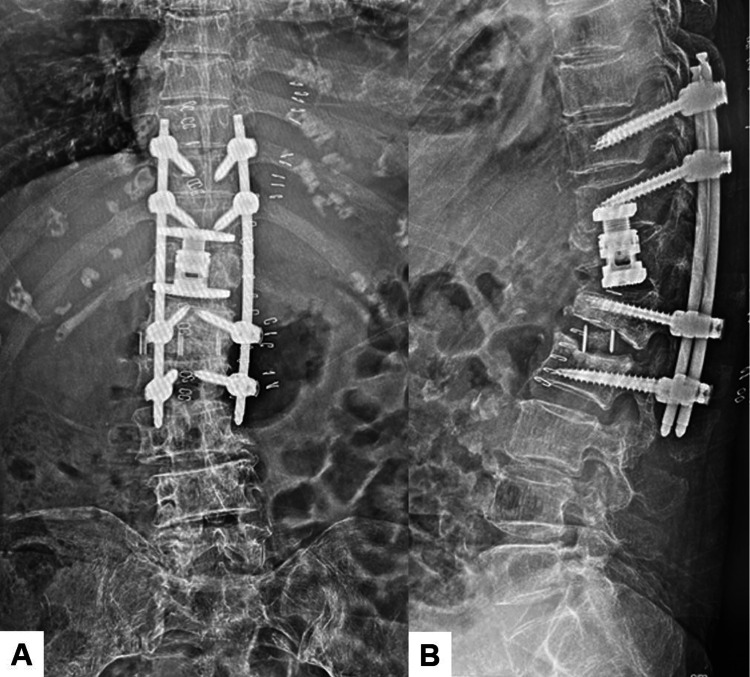
Postoperative images A: Anteroposterior lumbar radiogram and B: Lateral lumbar radiogram.

**Figure 13:**
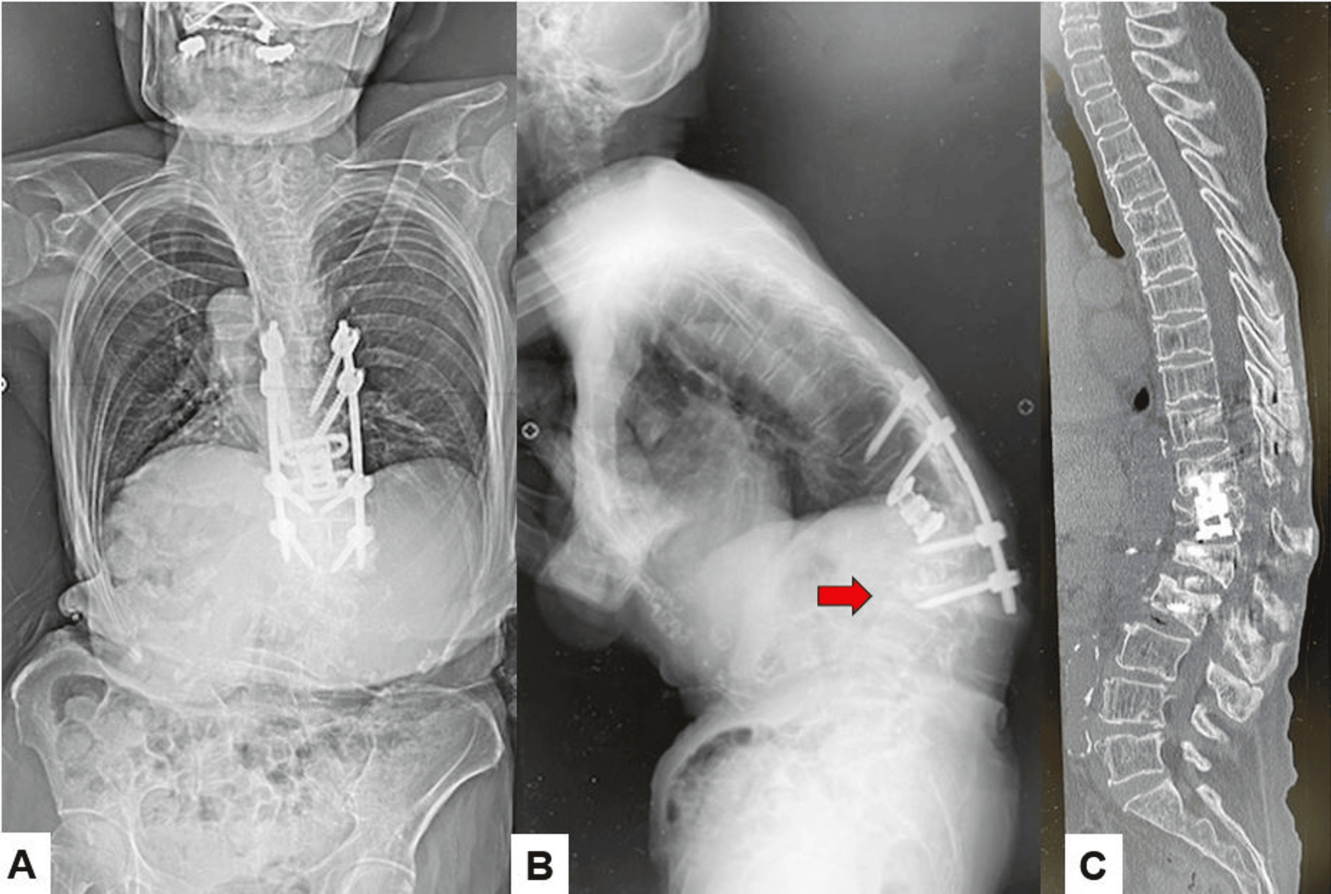
Follow-up images A: Posteroanterior whole spine radiogram, B: Lateral whole spine radiogram, and C: Mid-sagittal reconstruction CT. The red arrow indicates DJK due to a vertebral fracture. DJK: Distal junctional kyphosis

Case 2 presents a type 2 DJF, 82-year-old woman with T12 osteoporotic burst fracture. An 82-year-old woman was referred to our hospital due to severe low back pain. She cannot walk without support. She had round back and low back pain due to osteoporotic vertebral fractures of T12-L2. She cannot walk more than 100 meters but has no neurological deficits. Preoperative radiograms indicated thoracolumbar kyphoscoliosis. CT showed T12-L2 osteoporotic vertebral fractures (Figure 11). The patient underwent T12 corpectomy and T10-L2 posterior fusion with pedicle screws (Figure 12). The six-month follow-up image showed collapse of the L2 vertebra (Figure 13). 

Case 3

**Figure 14:**
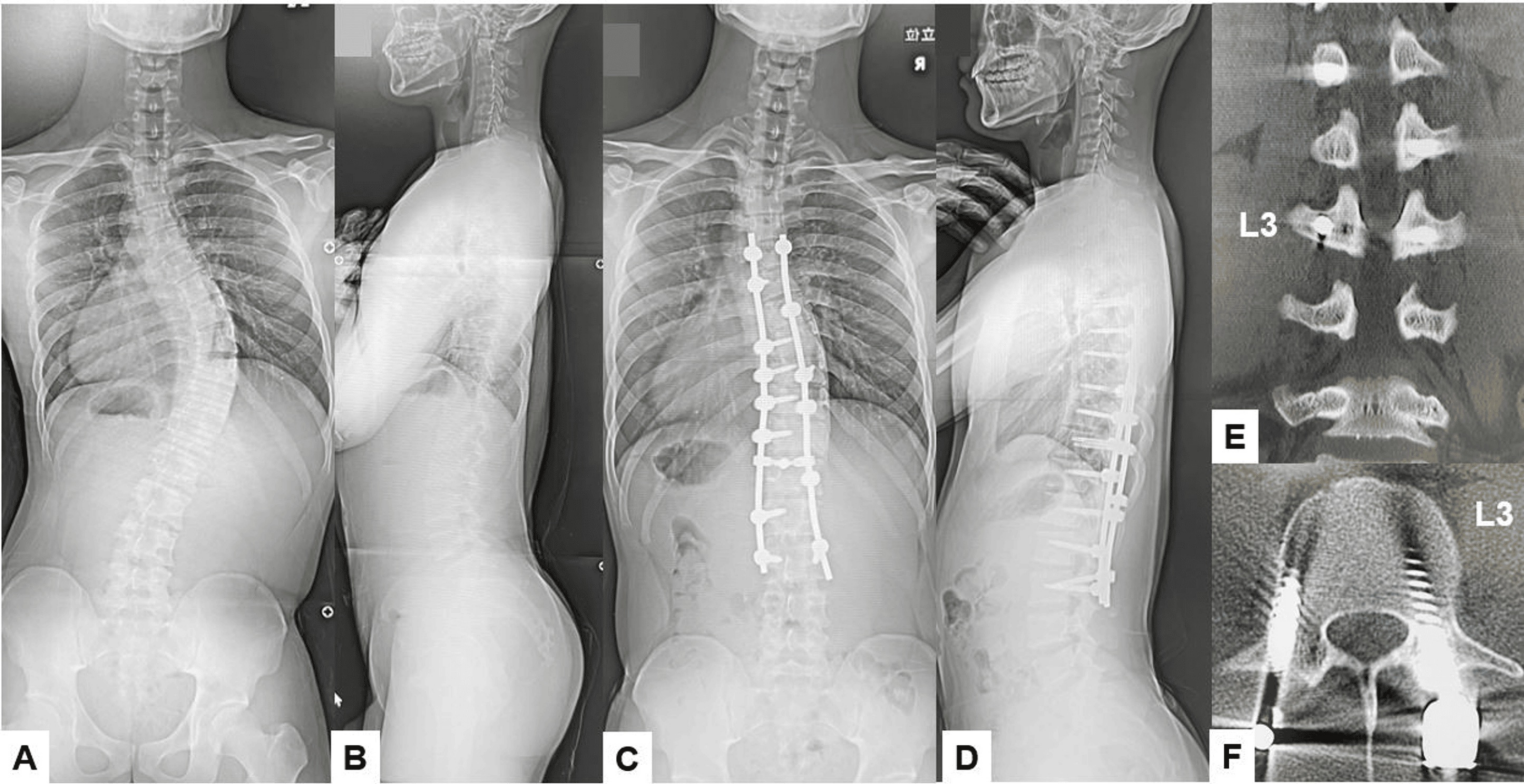
Pre-and postoperative images A: Preoperative posteroanterior whole spine radiogram, B: Preoperative lateral whole spine radiogram, C: Postoperative posteroanterior whole spine radiogram, D: Postoperative lateral whole spine radiogram, E: Coronal reconstruction lumbar CT, and F: Axial lumbar CT at L3.  The Cobb angle was corrected from preoperative 56 degrees to postoperative 23 degrees.

**Figure 15:**
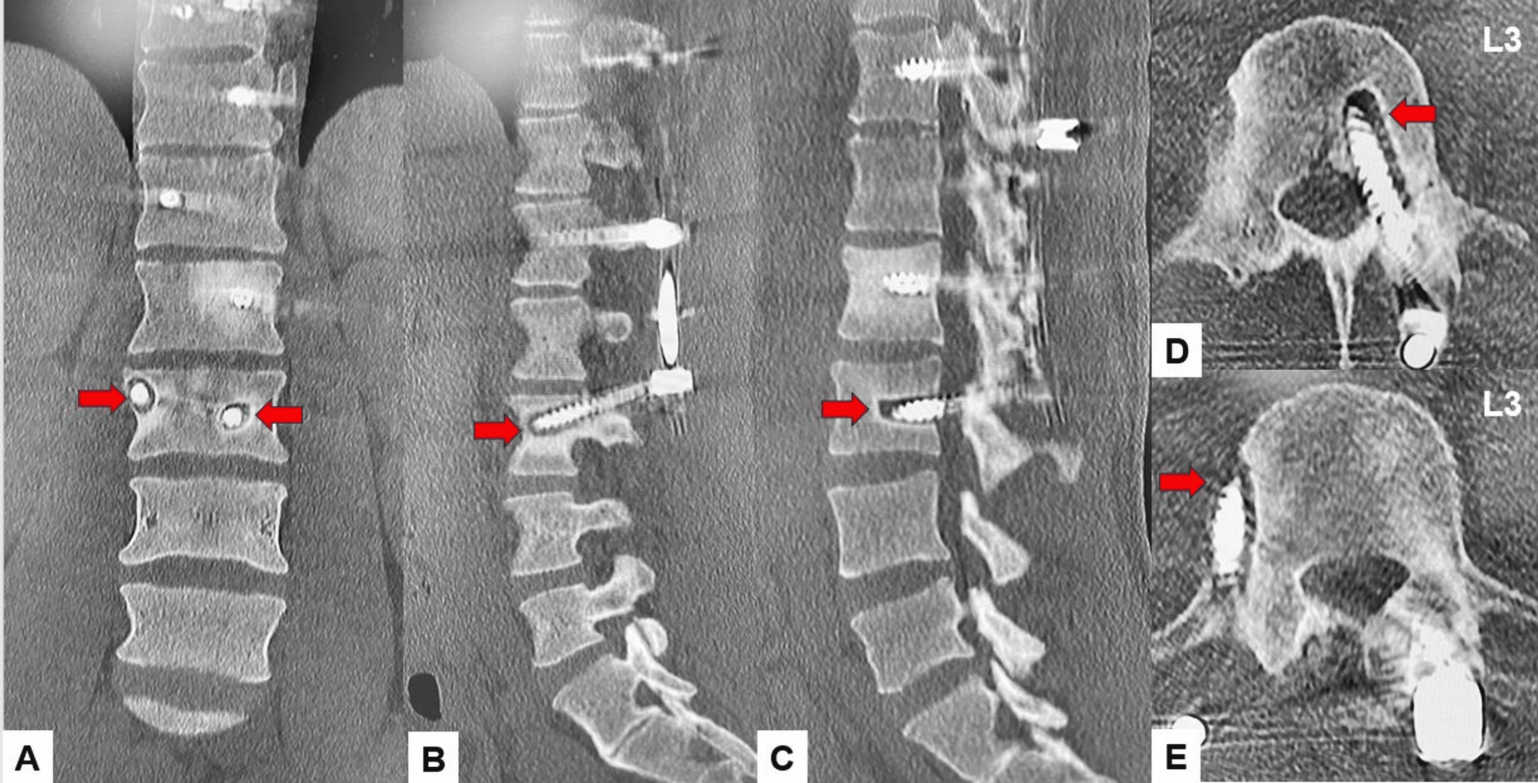
Five-year follow-up images A: Coronal lumbar reconstruction CT, B: Right parasagittal lumbar reconstruction CT, C: Left parasagittal lumbar reconstruction CT, and D and E: Axial CT at L3. Red arrows indicate screw loosening.

**Figure 16:**
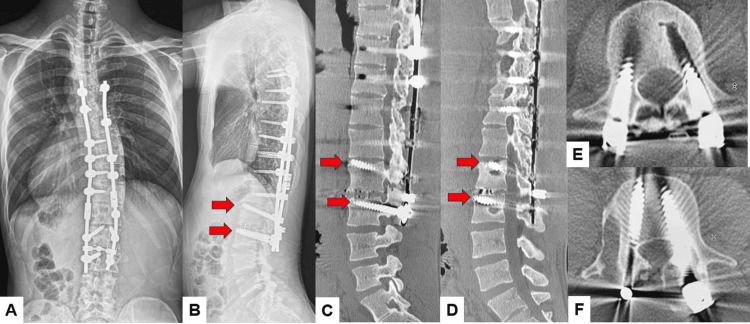
Postoperative images after revision surgery A: Postoperative posteroanterior whole spine radiogram, B: Postoperative lateral whole spine radiogram, C: Right parasagittal lumbar reconstruction CT, D: Left parasagittal lumbar reconstruction CT, and E and F: Axial CT at L3. Red arrows indicate transdiscal screws.

Case 3 presents a type 3 DJF, 22-year-old man, remnant of adolescent idiopathic scoliosis. A 22-year-old man was introduced to our hospital for scoliosis surgery. He had a spinal deformity from the age of 13, but no treatment was obtained. He had no neurological deficit. However, his rib cage protruded in the spinal flexed position, with a right-side rib hump of 3 cm. Preoperative radiograms showed right-sided thoracolumbar scoliosis. The Cobb angle was 56 degrees (Figures 14A, 14B). Postoperative whole spine radiogram indicated good spinal correction. The postoperative Cobb angle became 23 degrees (Figures 14C, 14D). L3 pedicle screws were inserted in adequate positions (Figures 14E, 14F). The patients experienced low back pain due to loosening of the L3 pedicle screws at the five-year follow-up (Figure 15). Revision surgery was performed with L2/3 oblique lateral interbody fusion (OLIF) and transdiscal screws. These transdiscal screws were across the previous screw hole and enhanced the screw pullout strength. Good bony fusion was obtained at one-year follow-up after revision surgery (Figure 16).

## Discussion

The progression of spinal fixation techniques has significantly improved patient outcomes, but challenges persist, particularly in DJF. DJF remains a critical complication following long-segment spinal fusions due to increased mechanical stress at the distal end, leading to kyphotic deformity, hardware failure, and vertebral fractures [9]. Richards et al. presented the earliest recorded instance of kyphosis developing at the distal junction between instrumented and non-instrumented spinal regions following segmental spinal instrumentation in their study of AIS treated with Cotrel-Dubousset instrumentation [10].

Although DJK is primarily recognized as a radiographic phenomenon, DJF is identified through clinical or radiographic signs of instability at the lower end of a spinal construct. This instability may result from multiple factors, including symptomatic DJK, mechanical failure of the construct such as rod fractures or screw displacement, pseudarthrosis, fractures, or adjacent segment degeneration [11].

DJF incidence varies based on underlying pathology and fusion techniques. In AIS patients, posterior fusion is associated with a higher DJF incidence (14.6%) compared to anterior fusion (7.1%), primarily due to differences in sagittal plane correction [1]. In ASD, fusion constructs terminating at L5 demonstrate a DJF incidence of 21.1%, with risk factors including advanced age, preoperative sagittal malalignment, and fusion length [12]. Osteoporotic thoracolumbar kyphosis presents an even higher DJF rate of 38.7%, often necessitating secondary pelvic fixation [13].

DJF is classified into three categories: Type 1: Pseudoarthrosis due to screw loosening/backout, which is typical of ASD; Type 2: fracture at the lower instrumented vertebra or one below which is relatively common for osteoporotic vertebra; and Type 3: Dislocation/angulation at the distal junctional area, which is mainly for AIS (Table 1). Addressing DJF often necessitates revision surgeries aimed at restoring spinal alignment, ensuring stability, and preventing recurrence. Various fixation techniques have been employed in these revision procedures, each with its advantages and limitations (Table 2).

**Table 1:** Three types of distal junctional failure

Type	Etiology	Typical case
1	Pseudoarthrosis due to screw loosening/backout	Adult spinal deformity
2	Fracture at the lower instrumented vertebra or one below	Osteoporotic vertebral fracture
3	Dislocation/angulation at the distal junctional area	Adolescent idiopathic scoliosis

**Table 2:** Comparative summary of various techniques

Technique	Advantage	Disadvantage
Transdiscal screw fixation	Enhances stability; useful in revision cases	Potential for disc damage; technically demanding
Cement-augmented screw fixation	Improves fixation in osteoporotic bone	Risk of cement leakage; potential thermal injury
Cortical bone trajectory (CBT)	Engages dense cortical bone; increases pullout strength	Limited applicability in severe deformities; learning curve
Larger diameter screw usage	Enhances mechanical stability	Requires sufficient bone stock; potential for pedicle fracture
Longer segment fixation	Redistributes mechanical stress; reduces implant failure risk	Increased surgical time; potential for reduced spinal mobility

Cement-augmented screw fixation

Cement augmentation involves the injection of polymethylmethacrylate or other bone cements into the vertebral body prior to screw insertion. In cases of severe bone loss, cement augmentation and vertebral body tethering enhance fixation strength [7]. Potential drawbacks include cement leakage, thermal injury, and difficulty in screw removal during future revisions.

Cortical bone trajectory (CBT)

The CBT technique utilizes a mediolateral and caudocranial screw path, maximizing contact with dense cortical bone. The CBT screw fixation offers superior pullout resistance and is particularly effective in osteoporotic patients [8]. However, it may not be suitable for all patients, particularly those with severe osteoporosis or complex deformities.

Larger diameter screws

Utilizing screws with a larger diameter can increase the surface area for the bone-screw interface, potentially enhancing fixation strength [14]. While this approach may improve pullout strength, it also carries the risk of pedicle fracture, especially in patients with compromised bone quality.

Longer segment fixation

Extending fusion to the sacrum or pelvis using iliac screws provides additional support and mitigates stress at the distal construct [14]. This technique distributes mechanical loads over a greater area, reducing stress on individual screws and enhancing overall construct stability. However, it increases the extent of surgery and the potential for blood loss and may impact spinal mobility.

Transdiscal screw fixation

Transdiscal screw fixation involves the insertion of screws across the intervertebral disc space, typically from the sacrum into the L5 vertebral body. This technique offers enhanced biomechanical stability by providing rigid fixation across the motion segment. However, it is technically demanding and carries risks such as nerve root injury and potential disruption of the disc space [15].

O-arm navigated transdiscal screw fixation: a novel approach

The novel technique of transdiscal screw fixation provides an alternative to extensive distal fusion by anchoring screws across the intervertebral disc space into the adjacent vertebrae. This method enhances biomechanical stability, addressing the challenge of compromised pedicle screw purchase in previously instrumented vertebrae [16].

O-arm navigated transdiscal screw fixation offers several advantages. The use of intraoperative 3D imaging ensures real-time guidance for precise screw placement across the disc space, reducing the risk of neural and vascular injury [17]. Biomechanical studies have shown that transdiscal screw fixation offers superior stability compared to conventional pedicle screws, with transdiscal screws demonstrating 1.6-1.8 times greater stiffness in all tested loading modes [18]. Furthermore, O-arm navigation has been associated with improved accuracy, reduced operative time, and decreased revision rates [19].

The adoption of O-arm guided transdiscal screw fixation with OLIF has significant clinical implications. This technique enhances construct stability, particularly in high-load-bearing regions, and reduces the likelihood of DJF recurrence. This technique also increases accuracy in screw placement, mitigating the risks associated with traditional revision methods. Additionally, OLIF enhances solid bony fusion in this type of revision surgery. As spinal fixation strategies continue to evolve, this novel approach may become a preferred technique for complex revision spine surgeries.

There are several limitations to this technique. The follow-up period of the cases is relatively short, and the number of cases is small. This technique carries potential risks, including screw perforation of the pedicle or pedicle fracture. Navigation is preferable for this technique.

## Conclusions

The adoption of O-arm guided transdiscal screw fixation has significant clinical implications. This technique enhances construct stability, particularly in high-load-bearing regions, and reduces the likelihood of DJF recurrence.

This innovative method also increases accuracy in screw placement, mitigating the risks associated with traditional revision methods, and preserves motion segments. As spinal fixation strategies evolve, this novel approach may become a preferred technique for complex revision spine surgeries.
